# Insights from the Molecular Modelling and Docking Analysis of AIF-NLS complex to infer Nuclear Translocation of the Protein

**DOI:** 10.6026/97320630014132

**Published:** 2018-03-31

**Authors:** Akash Srivaths, Shyam Ramanathan, Seethalakshmi Sakthivel, SKM Habeeb

**Affiliations:** 1 Department of Genetic Engineering, School of Bioengineering, SRM University, Kattankulathur, Chennai - 603203

**Keywords:** Apoptosis Inducing Factor (Mitochondrial) 1 (AIFM1), Apoptosis Inducing Factor Protein (AIF), Nuclear Localization Signal (NLS), Mitochondrial Localization Signal (MLS), Threading, Protein - Protein Interaction & Importin

## Abstract

Apoptosis Inducing Factor protein has a dual role depending on its localization in mitochondrion (energy production) and nucleus (induces apoptosis). Cell damage transports this protein to nucleus which otherwise favors mitochondrion. The alteration of Nuclear Localisation Signal tags could aid nuclear translocation. In this study, apoptosis inducing factor protein (AIF) was conjugated with strong NLS tags and its binding affinity with Importin was studied using in silico approaches such as molecular modeling and docking. This aims to improve the docking affinity of the AIF-Importin complex thus allowing for nuclear translocation, in order to induce caspase-independent apoptosis of the cell.

## Background

Cancer is the uncontrolled growth of abnormal cells that are invasive and destroy body tissues. It has become one of the most dreaded diseases in the recent times. In 2015, about 8.8 million people died due to cancer [1]. Treatment for any cancer generally includes a combination of chemotherapy, radiotherapy and surgery. These therapies suffer from a lack of specificity as they may kill normal cells as well, leading to lethal side effects [2].

Cancerous cell death can be induced using a protein, Apoptosis inducing factor 1, encoded by the AIFM1 gene located on the Xchromosome. The protein can localize to the mitochondria (for energy production and subsequent cell growth) as well as the nucleus (inducing caspase independent apoptosis) [3, 4, 5]. However, owing to its weak Nuclear Localization Signal (NLS), the protein does not localize to the nucleus except in response to apoptotic stimuli, preferring to carry out its mitochondrial function [6].

NLS is a monopartite or bipartite signal rich in positively charged amino acid residues (Lysine and Arginine residues) that tags protein for import into the nucleus [7]. NLS is recognized by Importin, a type of Karyopherin, which is involved in transporting proteins into the nucleus [8]. Importin consists of α and β subunits. Importin α is an adaptor protein that recognises and binds to the NLS of a nuclear protein [9]. The Importin α- NLS complex then proceeds to bind to Importin β, by means of Importin β binding domain (IBB), a 44-amino acid long sequence which is present at the N-terminus of the importin-α [10].

The binding with Importin simply facilitates its movement across the Nuclear Pore Complex (NPC), and once this is complete the Importin-NLS complex dissociates with the binding of Ran-GTP. This allows for the release of the NLS and thus the protein, and Importin is captured once again by the NPC, recycled and used for the transport of further proteins [11, 12]. The nuclear transport of the Apoptosis-inducing factor protein is not facilitated unless a DNA-damaging event occurs [13, 14]. On occurrence of such an event, the Apoptosis-inducing factor protein, is released from mitochondria following mitochondrial outer membrane permeabilization thereby inducing a caspaseindependent cell death [15, 16, 17, 18]. Moreover, AIF protein lacks a strong NLS, which prevents it from localizing into the nucleus.

This study aims at modifying the NLS tag of the AIF protein such that it aids in the translocation of the protein to the nucleus by taking precedence over its mitochondrial function.

## Methodology

### AIF Protein

The AIF protein was selected solely on the basis of the fact that it contains a Mitochondrial Localisation Signal (MLS) and an NLS, and the reason for its duality was due to the MLS being stronger than the NLS. This led to the reasoning that if the protein were reinforced with a stronger NLS tag, it would cause the protein to relocate to the nucleus after synthesis and thus induce apoptosis. The sequence of Apoptosis Inducing Factor (AIF) protein was retrieved from the Uniprot database holding an accession number O95831.

### Identification of NLS sites

A Nuclear Localization Signal would be present in all nuclear proteins; such proteins were obtained from the Nuclear Protein Database version.2.1 [19]. Over 3000 proteins were present in the database [20, 21, 22]. All of them were screened for the presence of an NLS sequence using the NLS Mapper [23]. The Mapper evaluates the NLS sequences by assigning a score for every residue, depending on its contribution towards the nuclear localization activity, and the cumulative score is totalled and displayed as the NLS score [24, 25, 26]. An NLS score of 8 and above signifies a strong signal, therefore it was chosen as the cut-off. Since more than 150 such NLSs were obtained, so the cut-off was raised to 15.

### Conjugation of NLS with AIF protein

**Figure 1:**
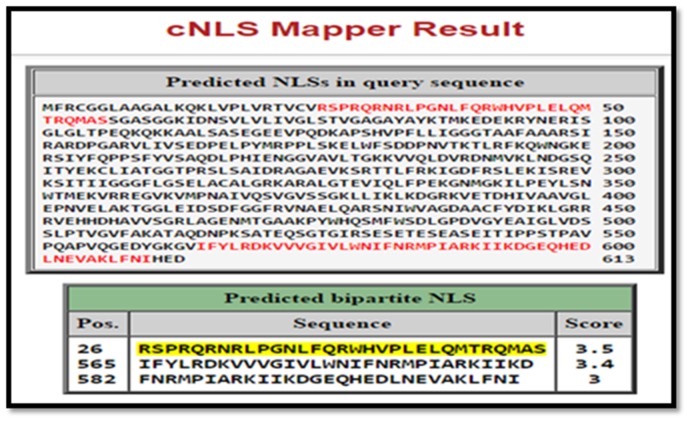
List of highest scoring NLSs in AIF protein. The highlighted regions in red are the NLS sequences present in the AIF protein. The highlighted sequence in yellow is the highest scoring NLS tag present

The best NLS tag of the AIF protein was present from amino acid position 26 to 56, which had a score of 3.5 (Figure 1). This site was replaced with a stronger NLS tag, which was obtained from the proteins in the Nuclear Protein Database. Moreover fusing the NLS tag at this particular site also ensures that the existing Mitochondrial Localization Signal is interrupted. This was done by obtaining the information of both sequences in the FASTA format and editing the AIF protein by inputting the NLS sequence in place of the pre-existing tag.

The recombinant protein, labelled RecAIF was modelled based on threading approach due to lack of proper template structures using ITASSER (Iterative Threading ASSEmbly Refinement). ITASSER generates the 3-dimensional structure of protein using "fold recognition" and also provides other parameters such as RMSD value and C-score which can be used to choose the best model [27, 28]. ITASSER also provides the Gene Ontology terms, which predict the Molecular function, Biological process and Cellular component of the modelled protein [29].

### Validation of the results

Models obtained from ITASSER were evaluated based on phi-psi Ramachandran plot (RC plot) using the RAMPAGE server [30]. ProtParam analysis was also performed on the primary sequence using ExPasy server to compute various physicochemical properties such as instability index, energy values, estimated half-life and GRAVY (Grand Average of Hydropathy).

### RecAIF-Importin docking

In order to facilitate the nuclear transport of the RecAIF protein, it should form a stable complex with the importin α, which carries the protein across the nuclear pore complex. Therefore, the interaction between NLS of the recombinant protein and its corresponding binding site at the importin α should be studied. The NLS binding site is present in the importin α from position 142 to 238. ClusPRO [31, 32, 33, 34, 35] was used to perform protein-protein docking.

## Result and Discussion

### cNLS mapping

The NLS mapper revealed that there is an NLS for the AIF protein from the position 26 to 56. This NLS was chosen to replace with the NLSs obtained from the Nuclear Protein Database. This is because, a Mitochondrial Localization Signal (MLS) is present in the protein from position 1 to 30 [6] and replacing this sequence will render the MLS redundant. Therefore, it would enhance the prospects of the protein getting localized to the nucleus.

### List of NLS sites

The search for proteins with NLS sites against the Nuclear Protein database resulted in 16 proteins having 24 NLS sites with score greater than 15. The proteins "Histone-lysine Nmethyltransferase" and "NUT family member 1" have 3 NLS sites. Four proteins have 2 NLS sites and rest of proteins have only one NLS site resulting in a total of 24 NLS sites. The NLS score ranges from 15-24. NLSs from "NUT family member 1" had two highest NLS score of 24 and 21.6 shown in Table 1. The identified NLS sites were conjugated with the target protein between sites 26 to 56, thus replacing AIF protein's N-terminal NLS and MLS sites.

### Model Validation

Models for all the conjugated proteins were generated and were subjected to Ramachandran Plot and Physicochemical properties analysis. The most vital criteria for selection of a recombinant protein model include RMSD values, C-score, Energy values and the instability index. All these criteria were obtained using ProtParam and GRAVY analysis [36].

### RC plot Analysis

The viability check is done in terms of validation of the model, using the Ramachandran Plot, which was obtained via the RAMPAGE server. The cut-off for this is that at least 90% of the recombinant protein's residues should be present within the favoured and allowed regions. Barring model ID 7.2, all the other models have their residues validated based on the cut-off provided by the Ramachandran Plot analysis, i.e., all other models have >90% of their residues within the allowed/favoured regions, implying that the protein's configuration with regards to the dihedral angles phi and psi are such that there is no steric hindrance regarding the protein's structure. This solves an important conundrum that may hinder protein-protein docking, and validates the structure based on the position of its residues and tells us the possible conformations of psi and phi angles for the amino acid residues of the protein. No models had >10% residues in the outlier region implying that all the models obtained had high viability and passed the validation.

### C-Score and RMSD Validation

The C-score is calculated based on the significance of threading template alignments and also on the parameter convergence of the structure assembly simulations. The C-score should be in the range of [-5, 2] for the model to be acceptable. As seen from Table 2, all the models satisfy this criterion, having C-scores ranging from -1.14 to -2.02. The RMSD values of all the structures in Table 2 are higher than expected, and hence it is unlikely that the recombinant AIF protein will fold in a similar manner to that of the actual AIF. The obtained RMSD values of ≈ 10±5 Å were all greater than the accepted 2.5±1 Å, suggesting that the recombinant protein may have a different fold to that of the native one.

### Energy of the Models

The energy of models spans between -16630.957 to -5429.86 kJ/mol (Table 2). In terms of energy values, only two structures with the lowest energy have been selected; i.e.; AIF conjugated with the NLS from NUT family member 1, with an energy value of -16630.957 kJ/mol and NLS score of 24 for NLS the sequence "RPSQPRKRRCDSFVTGRRKKRRRS". The second structure is AIF conjugated with the NLS from ATP-dependent RNA helicase DDX18, with an energy value of -15068.126. The NLS sequence is "KKKKKRKMVNDAEPDTKKAKTE".

### Validation by Instability Index

At first glance, it seems that the model containing the NLS from NUT family member 1 is superior because - 1) it has a lower energy score, and 2) it has a higher scoring NLS sequence. However, on checking the instability indices of all the given recombinant models, the model with the least instability index by quite a margin is model 16.1; i.e.; AIF conjugated with the NLS KKKKKRKMVNDAEPDTKKAKTE from ATP-dependent RNA helicase DDX18. Its instability index value is 46.43, as compared to that of model 7.1; i.e., AIF conjugated with NLS RPSQPRKRRCDSFVTGRRKKRRRS from NUT family member 1; which is 188.46. The instability index is used to measure the stability of the protein in a test tube. Protein with instability indices lesser than 40 is stable.

The instability indices of the recombinant models range from 46.43 to 295.9. The instability index of native protein is 48.27, which in itself is stable. Hence, this criterion was used to eliminate 90% of the models owing to their high instability indices. This was based on the fact that many naturally occurring proteins have instability indices of around 50, yet they are found to be stable in their native state. Thus, the cut-off was raised to 55 to ensure that only the best possible models get selected [37]. The model 16.1 was selected based on its low instability index of 46.43 close to native protein as opposed to model 7.1 with a value of 188.46. Thus, the protein models generated from the high scoring NLS tags of the protein "NUT family member 1" were not chosen due to the fact that their instability indices were extremely high (188.46 and 189.02) when compared to the model obtained using the NLS of the protein ATP-dependent RNA helicase DDX18 despite the fact that its NLS score is lower than that of the aforementioned protein. The data from all models regarding instability index, aliphatic index and GRAVY values can be seen in Table 3.

Thus, the recombinant model 16 was selected; containing the NLS sequence KKKKKRKMVNDAEPDTKKAKTE isolated from the protein ATP-dependent RNA helicase DDX18. This model was chosen because - 1) it had an acceptable C-score of -1.55, 2) it had the second lowest energy value -15068.126, 3) it had the lowest instability index of 46.43 close to native model, 4) When RAMPAGE analysis was performed, it was found that this model had the most number of residues in the favored region and second least number of residues in the outlier region (490 and 38 respectively) out of 24 models generated.

### Gene Ontology annotations

The GO terms for this recombinant protein seem to provide the most promising results since it has "Nucleotide binding" as one of its molecular functions, which happens to be an important factor for the apoptotic activity of the AIF. The GO terms "Establishment of localization" as its biological process shows that the protein will get localized with accordance to the signal peptide it carries (NLS in this case) and "Response to chemical stimulus" biological process shows that the localization process would alter the state of the cell (apoptosis in this case).

### Importin and Recombinant AIF Protein Docking

**Figure 3:**
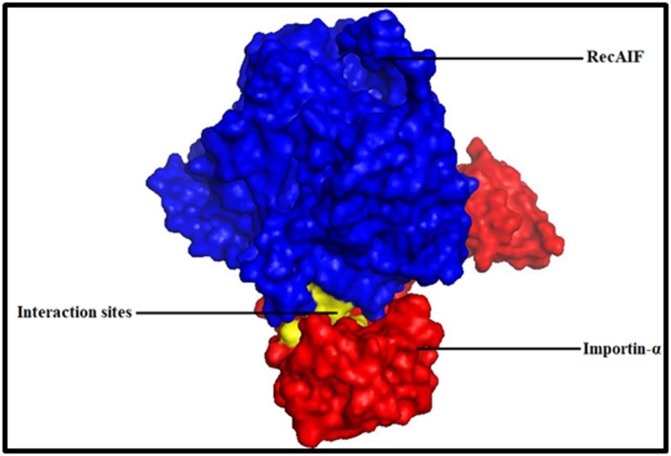
Surface model of Importin and Recombinant AIF Protein. Importin was shown in red color and Recombinant AIF in blue color. The interacting regions are in yellow color.

**Figure 4:**
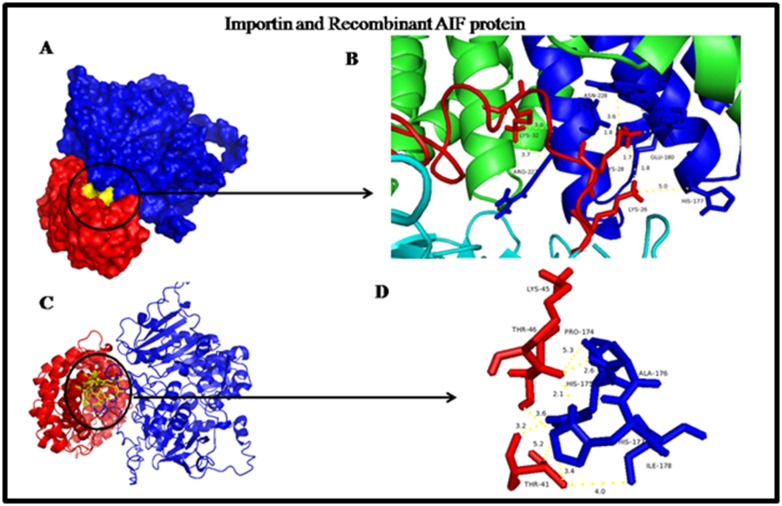
Docking interaction of Importin and Recombinant AIF protein. (A) Surface and (B & C) Cartoon representation. Importin was shown in blue colour and Recombinant AIF in red colour and the distance between the closest residues was shown in stick model in D.

Protein-protein interactions play a vital role in various aspects of the structural and functional organization of the cell, and a better understanding of cell processes such as metabolic control, signal transduction, and gene regulation. Molecular modeling approaches can be used to understand the details of proteinprotein interactions at the atomic level. ClusPro server, an FFT based algorithm was used to study the interaction between two proteins. ClusPro clusters and filters the docked complexes. Totally, 110 clusters were generated by from four methods namely; (i) balanced, (ii) Electrostatic favoured, (iii) hydrophobic favoured and (iv)VdW+Elec, each method gave 29, 29, 23 and 29 clusters respectively. The lowest energy values of the docked complex ALF-Importin model from balanced, electrostatic favoured, hydrophobic-favoured and VdW+Elec has -998.3, - 111.6, -1219.2 and -317.3 respectively. Best-docked complexes were analyzed manually to identify the possible interaction sites. The residues A222, C223, G224, E180, W184 and R227 were known to be involved in binding interaction. The complex 002.29 has major group of interacting residues from NLS binding site. The residues H177, E180, S219, L221, A222, C223, G224 and Y225 are known to be part of NLS binding site. The surface model of Importin and Recombinant AIF is shown in Figure 3. The binding pocket and NLS site residues were shown in yellow color and the details of interacting residues were given in cartoon representation in Figure 4. Total accessible surface areas of interacting residues between Importin and Recombinant AIF protein were given in Table 4.

## Contribution

The interaction of AIF-Importin complex was enhanced by the addition of an NLS from ATP-dependent RNA helicase, thus leading to nuclear translocation being favoured over mitochondrial translocation.

## Conclusion

From the above results, it is evident that the recombinant protein arising from the fusion of NLS of the ATP-dependent RNA helicase to the AIF protein has considerably low values for Instability Index, RMSD and Energy. The GO term "Establishment of localization" as its biological process shows that the protein will get localized to the nucleus owing to the NLS tag it carries. By performing protein-protein docking, it is also seen that the NLS in the recombinant AIF protein interacts with its binding site at the importin α. Therefore, it can be concluded that the NLS of the protein ATP-dependent RNA helicase is the best NLS tag for the AIF protein to enhance its interaction with Importin. This will aid in facilitating nuclear translocation over mitochondrial translocation.

## Figures and Tables

**Table 1:** List of NLS sites and the protein they were obtained from

LIST OF NLS SEQUENCES					
ID	Name of the Protein	Gene	Protein Length	NLS Sequence	Score
1.1	Chromodomain-helicase-DNA-binding protein 1-like	CHD1L	897	TKRKRVLSPEELEDRQKKRQEA	15
2.1	Activity-dependent neuroprotector homeobox protein	ADNP	1102	PVKRTYEQMEFPLLKKRKLD	18.2
3.1	activating transcription factor 7-interacting protein 1	ATF7IP	1270	EFSRRKRSKSEDMDNVQSKRRRY	16.3
4.1	Protein polybromo-1	PBRM1	1689	GPSRKRRRLS	18
4.2				PSRKRRRLS	17
5.1	Histone-lysine	EHMT2	1210	VKPSRKRRKRE	15
5.2	N-methyltransferase			PSRKRRKREP	15
5.3				RRKAKKKWRKDSPWVKPSRKRRKRE	15.5
6.1	Probable global transcription activator SNF2L2	SMARCA2	1590	KKRKRRRNVD	16
7.1	NUT family member 1	NUTM1	1132	RPSQPRKRRCDSFVTGRRKKRRRS	24
7.2				RPSQPRKRRCDSFVTGRRKKRRRSQ	21.6
7.3				QPRKRRCDS	15
8.1	mRNA-capping enzyme	RNGTT	597	RKHHLDPDTELMPPPPPKRPRP	16.9
9.1	Nuclear cap-binding protein subunit 1	NCBP1	790	MSRRRHSDENDGGQPHKRRKTS	15.4
9.2				SRRRHSDENDGGQPHKRRKTS	15.3
10.1	general transcription factor II-I repeat domain- protein 1	GTF2IRD1	976	PKRKRKRVS	15
10.2				PKRKRKRVSE	17
11.1	G1/S-specific cyclin-E1	CCNE1	2142	RSRKRKANVT	15
12.1	Death effector domain-containing protein	DEDD	318	SKRPARGRATLGSQRKRRKS	15.4
13.1	DNA (cytosine-5)-methyltransferase 3A	DNMT3A	912	RPGRKRKHPPV	18.5
14.1	BRCA2-interacting transcriptional repressor EMSY	EMSY	1322	EKPRKRRRTNS	16
15.1	histone-lysine N-methyltransferase EHMT1	EHMT1	1298	IKPARKRRRRS	16
15.2				PARKRRRRSR	17
16.1	ATP-dependent RNA helicase DDX18	DDX18	670	KKKKKRKMVNDAEPDTKKAKTE	16

**Table 2:** C-score, RMSD and Energy values for all the models from ITASSER analysis.

ITASSER RESULTS						
ID	Protein Name	NLS Sequence	Score	C-SCORE	RMSD (in Å)	Energy (kJ/mol)
1.1	Chromodomain-helicase-DNA-binding protein 1-like	TKRKRVLSPEELEDRQKKRQEA	15	-1.68	11.8±4.5	-15091.765
2.1	Activity-dependent neuroprotectorhomeobox protein	PVKRTYEQMEFPLLKKRKLD	18.2	-1.69	11.84±.5	-7689.002
3.1	Activating transcription factor 7-interacting protein 1	EFSRRKRSKSEDMDNVQSKRRRY	16.3	-1.89	12.3±4.4	-13883.137
4.1	Protein polybromo-1	GPSRKRRRLS	18	-1.25	10.6±4.6	-11742.25
4.2		PSRKRRRLS	17	-1.25	11.2±4.6	-6694.036
5.1	Histone-lysine N-methyltransferase	VKPSRKRRKRE	15	-1.57	11.4±4.5	-13415.11
5.2		PSRKRRKREP	15	-1.62	11.6±4.5	-8035.358
5.3		RRKAKKKWRKDSPWVKPSRKRRKRE	15.5	-1.79	12.1±4.4	-13267.012
6.1	Probable global transcription activator SNF2L2	KKRKRRRNVD	16	-2.08	11.4±4.5	-11896.704
7.1*	NUT family member 1	RPSQPRKRRCDSFVTGRRKKRRRS	24	-1.92	12.4±4.3	-16630.957
7.2		RPSQPRKRRCDSFVTGRRKKRRRSQ	21.6	-2.02	12.7±4.3	-7788.716
7.3		QPRKRRCDS	15	-1.72	11.8±4.5	-11836.411
8.1	mRNA-capping enzyme	RKHHLDPDTELMPPPPPKRPRP	16.9	-1.97	12.5±4.3	-11266.314
9.1	Nuclear cap-binding protein subunit 1	MSRRRHSDENDGGQPHKRRKTS	15.4	-2	12.6±4.3	-9159.591
9.2		SRRRHSDENDGGQPHKRRKTS	15.3	-2	11.3±4.5	-16514.232
10.1	general transcription factor II-I repeat domain protein-1	PKRKRKRVS	15	-1.39	11.0±4.6	-11939.963
10.2		PKRKRKRVSE	17	-1.92	12.3±4.3	-5429.863
11.1	G1/S-specific cyclin-E1	RSRKRKANVT	15	-1.37	10.9±4.6	-10473.442
12.1	Death effector domain-containing protein	SKRPARGRATLGSQRKRRKS	15.4	-1.89	12.3±4.4	-12811.495
13.1	DNA (cytosine-5)-methyltransferase 3A	RPGRKRKHPPV	18.5	-1.54	11.4±4.5	-13727.989
14.1	BRCA2-interacting transcriptional repressor EMSY	EKPRKRRRTNS	16	-1.52	11.3±4.5	-8029.218
15.1	histone-lysine N-methyltransferase EHMT1	IKPARKRRRRS	16	-1.14	10.4±44.6	-7391.603
15.2		PARKRRRRSR	17	-1.51	11.3±44.5	-12115.136
16.1*	ATP-dependent RNA helicase DDX18	KKKKKRKMVNDAEPDTKKAKTE	16	-1.55	11.4±44.5	-15068.126
* Model with lowest energy value.						

**Table 3:** Instability Index, Aliphatic Index and GRAVY values for the selected models.

ProtParam Analysis							
ID	Mol.Wt.	Theoretical PI	Negative Residues	Positive Residues	Instability Index	Aliphatic Index	GRAVY
1.1	2725.1	9.98	5	8	128.28	53.18	-2.164
2.1	2520.03	9.83	3	6	57.29	73	-1.175
3.1	2959.3	10.9	4	9	172.68	12.61	-2.404
4.1	1212.42	12.48	0	5	194.68	39	-2.17
4.2	1155.37	12.48	0	5	215.2	43.33	-2.367
5.1	1439.73	12.01	1	7	145.98	26.36	-2.855
5.2	1309.54	12.01	1	6	189.26	0	-3.33
5.3	3291.95	12.14	2	15	113.48	15.6	-2.812
6.1	1355.61	12.01	1	7	186.07	29	-3.25
7.1	3014.52	12.3	1	12	188.46	12.08	-2.35
7.2	3142.65	12.3	1	12	189.02	11.6	-2.396
7.3	1145.3	10.76	1	4	164.58	0	-2.7
8.1	2612.05	9.98	3	5	91.93	35.45	-1.918
9.1	2635.86	11.44	3	7	165.08	0	-2.627
9.2	2504.67	11.44	3	7	151.54	0	-2.843
10.1	1154.43	12.31	0	6	117.56	32.22	-2.6
10.2	1283.54	11.73	1	6	126.06	29	-2.69
11.1	1215.42	12.31	0	5	96.31	39	-2.03
12.1	2296.67	12.7	0	9	95.39	29.5	-2.015
13.1	1327.6	12.31	0	5	88.64	26.36	-2.318
14.1	1427.63	12.01	1	6	115.5	0	-3.264
15.1	1423.73	12.6	0	7	237.83	44.55	-2.4
15.2	1338.59	12.7	0	7	295.9	10	-3.15
16*	2602.09	10.1	4	10	46.43	22.27	-2.291
*Selected model with lowest instability index.							

**Table 4:** Accessible Surface Area of RecAIF residues found to be interacting with importin-α.

ASA Analysis of interacting residues of RecAIF								
Probe radius: 1.400 (Default value of water probe)								
S.No.	Residue	Residue no.	Total accessible surface	Apolar	Backbone	Sidechain	Ratio	In/Out
1	HIS	177(RecAIF)	106.8	85.84	2.93	103.44	66.9	Out
2	GLU	180(RecAIF)	46.77	6.23	0	46.77	33.1	-
3	SER	219(RecAIF)	75.89	39.36	25.6	50.3	65	Out
4	LEU	221(RecAIF)	45.66	39.05	9.29	36.38	24.9	-
5	ALA	222(RecAIF)	74.34	72.02	18.74	55.59	85.7	Out
6	CYS	223(RecAIF)	75.18	19.5	2.54	72.65	71	Out
7	GLY	224(RecAIF)	45.11	38.38	45.11	0	51.7	Out
8	TYR	225(RecAIF)	17.32	15.15	0.13	1.19	8.9	In
9	TRP	184(RecAIF)	78.85	65.4	0	78.85	35.1	-
10	ARG	227(RecAIF)	66.04	27.91	2.79	63.25	32.4	-
All values provided (except for ratios) are in Å2. Out and In refer to exposed/buried residues.								

**Figure 2:**
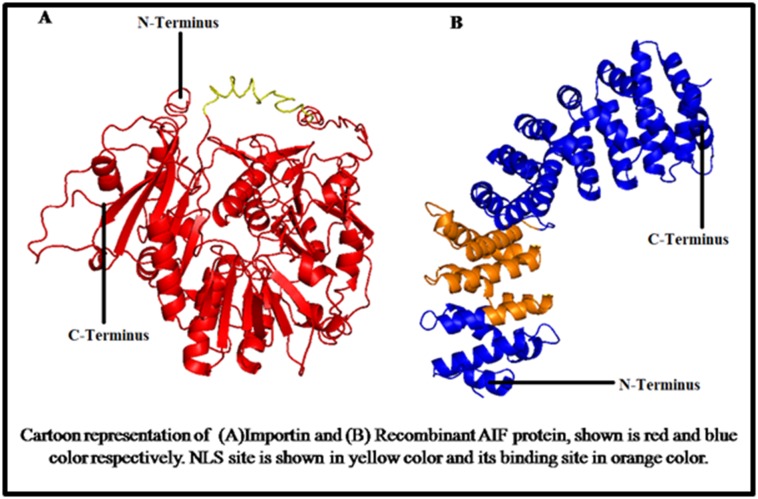
Cartoon representation of (A) Recombinant AIF protein and (B)Importin, shown is red and blue colour respectively. NLS site is shown in yellow colour and its binding site in orange colour.
